# *Helicobacter pylori* plasticity region genes are associated with the gastroduodenal diseases manifestation in India

**DOI:** 10.1186/s13099-016-0093-5

**Published:** 2016-03-22

**Authors:** Mou Ganguly, Sagartirtha Sarkar, Prachetash Ghosh, Avijit Sarkar, Jawed Alam, Bipul Chandra Karmakar, Ronita De, Dhira Rani Saha, Asish K. Mukhopadhyay

**Affiliations:** Division of Bacteriology, National Institute of Cholera and Enteric Diseases, P 33, CIT Road, Scheme XM, Beliaghata, Kolkata, 700010 India; Department of Zoology, University of Calcutta, Kolkata, India

**Keywords:** *Helicobacter pylori*, Plasticity region, Duodenal ulcer, IL-8, Apoptosis

## Abstract

**Background:**

Almost all *Helicobacter pylori* infected person develop gastritis and severe gastritis is supposed to be the denominator of peptic ulcer diseases, which may lead to gastric cancer. However, it is still an enigma why few strains are associated with ulcer formation, while others are not related with any disease outcome. Although a number of putative virulence factors have been reported for *H. pylori*, there are contradictory results regarding their connotation with diseases. Recently, there has been a significant attention in strain-specific genes outside the *cag* pathogenicity island, especially genes within plasticity regions. Studies demonstrated that certain genes in this region may play important roles in the pathogenesis of *H. pylori*-associated diseases. The aim of this study was to assess the role of selected genes (*jhp0940*, *jhp0945*, *jhp0947* and *jhp0949*) in the plasticity region in relation to risk of *H. pylori*-related diseases in Indian population.

**Methods:**

A total of 113 *H. pylori* strains isolated from duodenal ulcer (DU) (n = 61) and non-ulcer dyspepsia (NUD) subjects (n = 52) were screened by PCR and Dot-Blot to determine the presence of these genes. The comparative study of IL-8 production and apoptosis were also done by co-culturing the AGS cells with *H. pylori* strains of different genotype.

**Results:**

PCR and Dot-Blot results indicated that the prevalence rates of *jhp0940*, *jhp0945*, *jhp0947* and *jhp0949* in the *H. pylori* strains were 9.8, 47.5, 50.8, 40.9 % and 17.3, 28.8, 26.9, 19.2 % isolated from DU and NUD, respectively. IL-8 production and apoptotic cell death were significantly higher in *H. pylori* strains containing *jhp0945*, *jhp0947* and *jhp0949* than the strains lacking those genes. Results indicated that the prevalence of *jhp0945*, *jhp0947* and *jhp0949* are associated with increased risk of severe diseases in India.

**Conclusion:**

Our study showed that presence of *jhp0945*, *jhp0947* and *jhp0949* were significantly associated with symptomatic expressions along with the increased virulence during in vitro study whereas *jhp0940* seems to be negatively associated with the disease. These results suggest that *jhp0945*, *jhp0947* and *jhp0949* could be useful prognostic markers for the development of duodenal ulcer in India.

## Background

*Helicobacter pylori* is a Gram negative microaerophilic bacterium that infects more than 50 % of world population by selectively colonizing the human stomach [1]. Although most infections are asymptomatic, 10–15 % of *H. pylori* infected individuals develop chronic inflammation leading to atrophic gastritis, peptic ulcer as well as gastric adenocarcinoma and gastric mucosa-associated lymphoid tissue lymphoma (MALT) [2–4]. It may also contribute to childhood malnutrition and increase the risk or severity of infection by other gastrointestinal pathogens such as *Vibrio cholerae*, especially in developing countries. In India, around 65–70 % populations are infected with *H. pylori* [5, 6]. The conundrum of *H. pylori* study is that infection remains latent in majority of the infected patients, while only approximately 15–20 % of infected individuals become symptomatic for peptic ulcer (duodenal or gastric) as a long-term consequence of infection. Infection usually starts in early childhood and the bacteria have a unique capacity to live in gastric milieu for lifelong unless eradicated by specific antibiotic treatment. It is still unclear what determines the outcome of an infection and the apparent paradox suggests that mere presence of *H. pylori* in the stomach is insufficient to cause gastric disease, rather requiring additional conditions. However, it is thought to involve interplay between the virulence of the infecting strain, host genetics and environmental factors. Experience with other bacterial pathogens suggests that *H. pylori*-specific factors may exist that influence the pathogenicity of *H. pylori.*

*H. pylori* bear an arsenal of specific virulence factors. Among them the cytotoxin-associated gene-pathogenicity island (*cag*-PAI), vacuolating associated cytotoxin gene A (*vacA*), outer inflammatory protein A (*oipA*), blood group antigen binding adhesin (*babA*), lipases and lipopolysaccharides (LPS) are potentially toxigenic to initiate the process of inflammation in the host gastric tissues and have been studied in great details to understand their association with gastroduodenal diseases. The gene that encodes CagA is part of a ~40 kb horizontally acquired DNA segment in the *H. pylori* genome known as *cag*-PAI [7]. *cagA* was the first reported gene in *H. pylori* strains that considered as a marker for the presence of *cag*-PAI, which include a number of other genes associated with increased virulence [8]. The *cag*-PAI also contain genes encoding a type IV secretion system, to ensure efficient translocation of the CagA protein into the host epithelium. One potential discordant that has complicated identification of certain disease-specific *H. pylori* virulence factors is the substantial geographic diversity in the prevalence of *H. pylori* virulence factors. Although presence of *cagA* is significantly associated with the disease status in Western countries, but in Asian countries (including Japan, China and India) this correlation was not observed as majority of the *H. pylori* strains in this region carry *cagA* gene [7, 9, 10].

Several studies reported the unusual genetic heterogeneity of *H. pylori* in terms of allelic diversity, which has established it as a species with a very high population recombination rate, and also enabled to identify the strains from various populations of different geographic regions [11]. Comparative analysis of the full genome sequences of two *H. pylori* strains (26695 and J99) indicated several regions whose G + C content was lower than that of the rest of *H. pylori* genome, indicating horizontal DNA transfer from other species. *H. pylori* contains an open pan-genome, in which each individual is found to possess distinct set of non-core, or strain-specific, genes [11]. On the basis of comparative analysis of the first sequenced *H. pylori* genomes, it can be said that these strain-specific genes are mostly found in genomic regions that had previously been coined as plasticity zones, a designation initially used to describe a particular genetic locus with high variation between the first two *H. pylori* genome sequences [11]. The availability of more sequencing data and more complete *H. pylori* genome sequences makes it clear that parts of the plasticity zones are generally organized as genomic islands that may be incorporated in one of quite a few different genetic loci. Approximately half of the strain-specific genes of *H. pylori* are positioned in the plasticity region [12]. For example, this plasticity region in strain J99 is continuous and 45 kb long whereas it is 68 kb discontinuous in strain 26695. Among the 38 open reading frames (ORFs) of the plasticity zone (*jhp0914*–*jhp0951)* in strain J99, only six are present in strain 26695 [13–17]. Although various representative genes of these plasticity regions have been recommended as disease markers, e.g. *dupA* for duodenal ulcer [18, 19], or *jhp950* for marginal zone B cell MALT lymphoma [20], the functions of the plasticity zones are still not clear yet. The different combinations of genes within plasticity regions are directly related to the variability of the gene content of *H. pylori* [21].

It is not clearly understood whether strain-specific genes or combinations of strain specific genes force the severity of gastric mucosal inflammation and the risk of various *H. pylori*-mediated diseases. Additionally, the functional importance of the majority number of open reading frames (ORFs) in the plasticity region remain unrevealed. Recently it has been reported that *jhp0940*, *jhp0945*, *jhp0947*, and *jhp0949* of Western *H. pylori* strains showed an association with an increased chance of gastroduodenal disease and an increase in inflammatory cytokines [15, 17, 22]. However, in other studies, the role of selected genes in the plasticity region in relation to the risk of *H. pylori*-related disease and the severity of gastric mucosal damage was debatable and uncertain. Furthermore, the reported associations need to be confirmed in other geographic regions, since geographic differences with regard to virulence genes of *H. pylori* have been demonstrated [16, 23, 24].

Indian *H. pylori* strains are genetically distinct than East Asian and Western strains [24]. Moreover, our recent study showed that presence of strains with intact *cag*-PAI was found more frequently in Kolkata than in Southern India indicating regional variation in the *H. pylori* gene pools [9]. In addition, India constitutes about 1/5th of the world’s population and there were no reports regarding the distribution of different plasticity genes and their correlation to disease from India except the *dupA* gene. These observations and our continuing interest in the dynamics of genetic traits associated with *H. pylori* infection and disease association motivated us to perform the present study for examining the prevalence of *jhp0940*, *jhp0945*, *jhp0947*and *jhp0949* of *H. pylori* and their relation to *H. pylori*-related disease in Indian population along with their role in in vitro study.

## Results

Among the enrolled 171 subjects suffering from gastroduodenal problems, a total of 113 *H. pylori* strains were isolated by culture method. Based on the visual examination of the stomach and duodenum during endoscopy, subjects were divided into two groups: non-ulcer dyspepsia (NUD) and duodenal ulcer (DU) patients. All the strains were isolated from these two groups: (1) 61 DU patients and (2) 52 NUD. Out of 61 DU cases, the mean age difference was 47.3 ± 9.8 and among 52 NUD subjects, the mean age difference was 31.6 ± 9.9. The genomic DNA from these 113 strains was used for further PCR based analysis.

### Distribution of *jhp0940*, *jhp0945*, *jhp0947* and *jhp0949* and their disease association

**Fig. 1:**
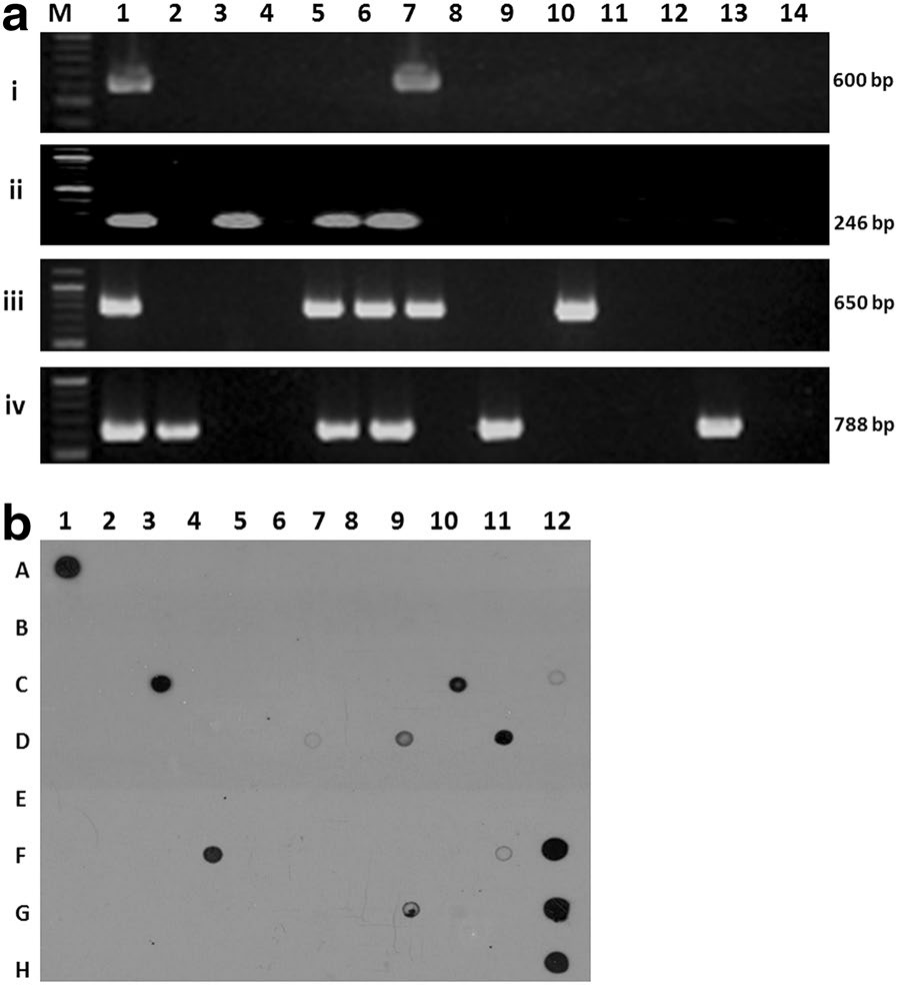
**a** Genotyping of plasticity region genes in Indian *H. pylori* isolates. The images shown are from a representative gel electrophoresis of a PCR amplification product of plasticity region genes from Indian isolates and J99 control strain with (1) jhp0940F and jhp0940R primers [22], (2) jhp0945F and jhp0945R primers [22], (3) jhp0947F and jhp0947R primers [22], and (4) jhp0949F and jhp0949R primers [22]. Where *lane 1*: DNA ladder *lane 2*: positive control, *lanes 3–13*: clinical isolates and *lane 14*: negative control. **b** Genotyping of *jhp0940* gene in Indian *H. pylori* isolates by dot blot hybridization assay. The images shown are from a representative dot blot hybridization of *jhp0940* gene from Indian isolates and J99 control strain (A1 and H12 represent as positive controls)

**Fig. 2:**
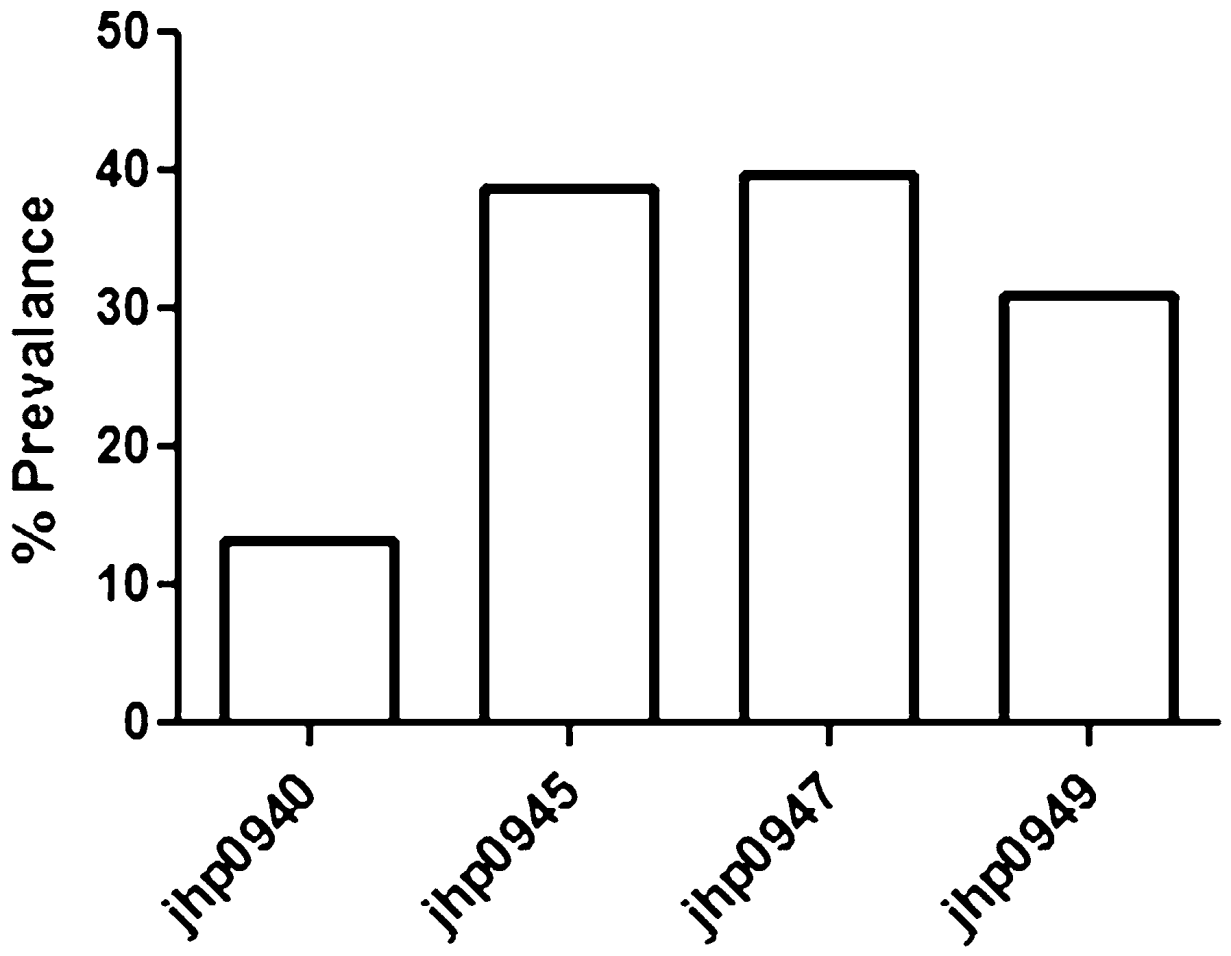
Distribution of *jhp0940*, *jhp0945*, *jhp0947*, and *jhp0949* genotypes among Indian *H. pylori* isolates

Prevalence of these selected genes in the plasticity region of *H. pylori* among NUD and DU patients in Indian population was screened by PCR and dot blot hybridization (Fig. 1a, b). All the strains yielded 480-bp product similar to *ureB* gene, indicating the identity of *H. pylori* DNA. All samples considered negative by PCR were confirmed as negative by dot blot hybridization. The prevalence rates of *jhp0940*, *jhp0945*, *jhp0947*, and *jhp0949* in patients with *H. pylori* were 13.3 % (15/113), 38.9 % (44/113), 39.8 % (45/113) and 31 % (35/113), respectively (Fig. 2). There was a low frequency of *jhp0940* gene in Indian population and 17.3 and 9.8 % strains were positive in NUD and DU patients, respectively (Table 1). *jhp0940* is almost 2 times higher in NUD than the DU even though difference was not significant.

**Table 1:** Prevalence of *jhp0940*, *jhp0945*, *jhp0947* and *jhp0949* genes in the plasticity region of Indian *Helicobacter pylori* isolates

No. of strains screened	*Jhp0940*	*Jhp0945*	*Jhp0947*	*Jhp0949*
NUD (52)	9 (17.3 %)	15 (28.8 %)	14 (26.9 %)	10 (19.2 %)
DU (61)	6 (9.8 %)	29 (47.5 %)	31 (50.8 %)	25 (40.9 %)
OR (95 % CI)	1.91 (0.63−5.80)	2.23 (1.02−4.88)	2.80 (1.27−6.19)	2.92 (1.23−6.87)
*P* value	0.130	0.042*	0.009*	0.013*

*OR* odds ratio, *CI* confidence interval

* Statistically significant

**Fig. 3:**
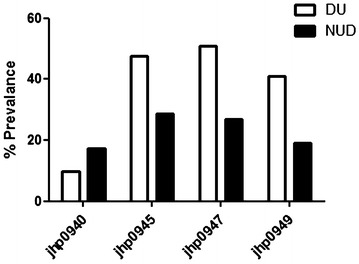
Prevalence of *jhp0940*, *jhp0945*, *jhp0947*, and *jhp0949* genotypes of Indian *H. pylori* strains isolated from DU patients and NUDs

*jhp0945* gene was found in 28.8 and 47.5 % of the strains isolated from NUD and DU patients, respectively indicating that *jhp0945* gene was significantly associated with DU than NUD (*P* = 0.042 and OR 2.23; 95 % CI 1.02–4.88) (Table 1). 14 (26.9 %) of the 52 patients with NUD and 31 (50.8 %) of the 61 patients with DU were colonized by a *jhp0947*-positive strain. In the univariate analysis, when patients with NUD and DU were compared, the presence of the *jhp0947*gene was positively associated with DU (*P* = 0.009; OR 2.80; 95 % CI 1.27–6.19). Similarly, *jhp0949* gene was detected in 19.2 and 40.9 % of the strains isolated from NUD and DU patients, respectively. Results demonstrated the significant association of *jhp0949* with DU than NUD (*P* = 0.013 and OR 2.92; 95 % CI 1.23–6.87). Among the 35 *jhp0949* positive strains, 33 were positive for *jhp0947* and 25 strains were also positive for all three elements *(jhp0945, jhp0947 and jhp0949).* So, the presence of *jhp0949* was completely linked with that of *jhp0947* in Indian population and was roughly associated with that of *jhp0945* (Fig. 3). In our study, only one strain containing all four ORFs (*jhp0940*, *jhp0945*, *jhp0947*, and *jhp0949*) was detected.

**Fig. 4:**
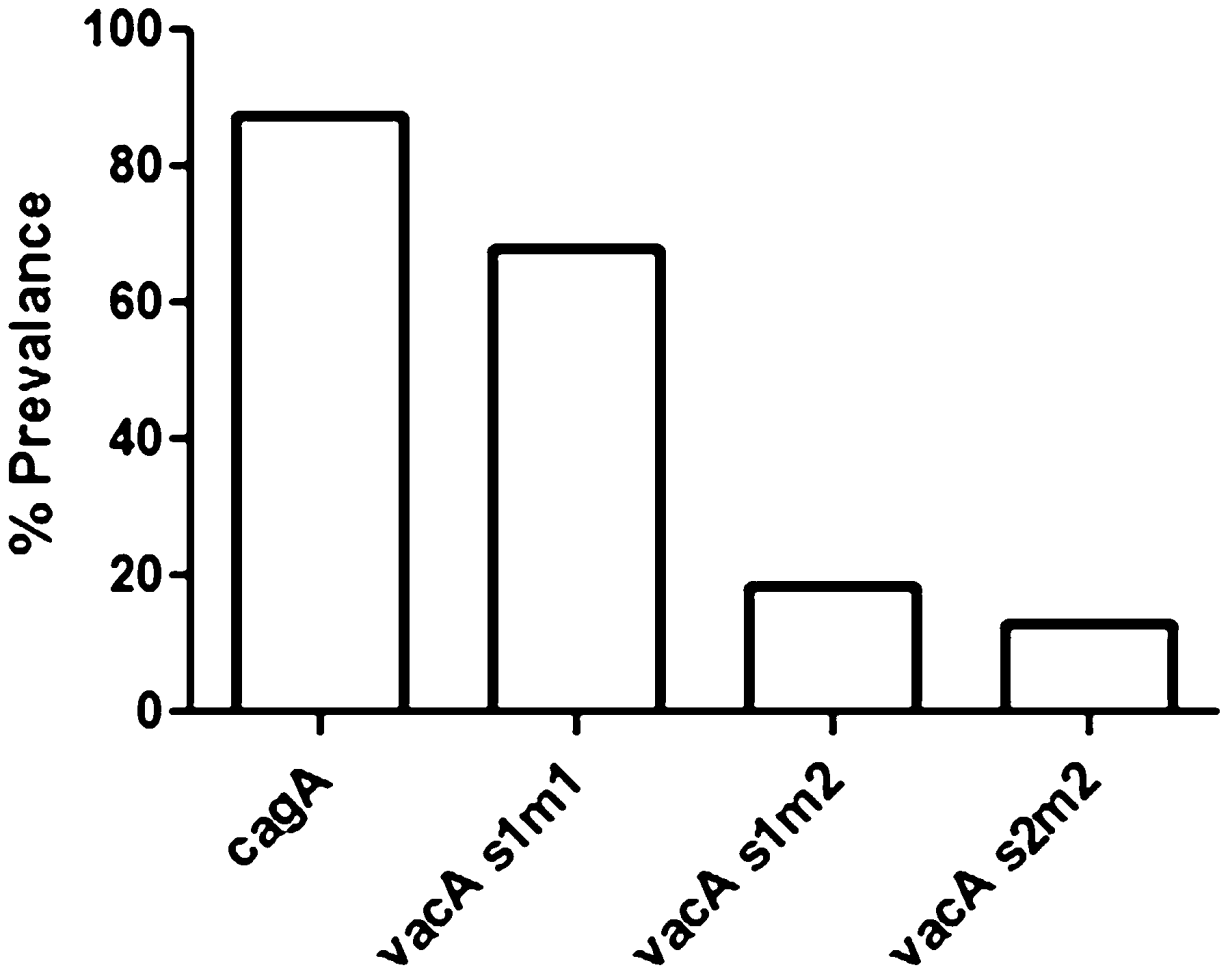
Distribution of *cagA* positive and different genotypes of *vacA* among Indian *H. pylori* isolates

The *cagA* and *vacA* status were determined using primers and protocols described earlier [23, 25]. *cagA* was present in 87.6 % (99/113) of the tested strains from this region. 68 % (77/113) of the strains had *vacAs1m1* allele. Other two alleles *s1m2* and *s2m2* of *vacA* were present in 18.6 % (21/113) and 13.3 % (15/113), respectively (Fig. 4). Status of *cagA* and *vacA* genes did not have any correlation with the presence of plasticity region genes indicating the presence of these two virulence-associated genes independent of the plasticity region genes.

### *jhp0945*, *jhp0947* and *jhp0949* positive strains showed greater IL-8 induction in gastric epithelial (AGS) cells

**Fig. 5:**
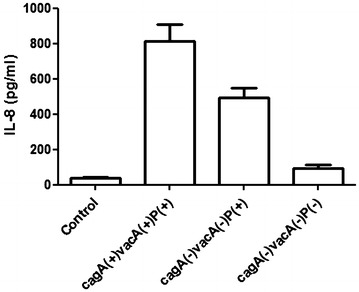
Role of *H. pylori* plasticity region in in vitro induction of IL-8. In vitro IL-8 production from AGS cells co-cultured with randomly selected *cagA*(+)*vacA*(+)P(+), *cagA*(−)*vacA*(-)P(+), and *cagA*(−)*vacA*(−)P(−) *H. pylori* strains (MOI is 100) for 8 h. IL-8 from culture supernatant was measured using ELISA as described in "Methods" section. Data are expressed as mean ± standard error of mean (SEM) of 3 experiments in duplicates. *vacA* positive and negative denote the *vacA* s1m1 and *vacA* s2m2 alleles, respectively

According to the presence of these virulence genes, the strains were divided into three groups viz. (1) triple positive strains (*cagA*^+^*/vacA*^+^*/*three ORFs^+^*(jhp0945*, *jhp0947*, and *jhp0949*), (2) only three ORFs positive strains (*cagA*^*−*^*/vacA*^−^*/*three ORFs^+^), and (3) triple negative strains (*cag*^−^*/vacA*^−^*/*three ORFs^−^). AGS cell were co-cultured with *H. pylori* strains for 8 h. IL-8 secretion was significantly (*P* < 0.05) higher from those cells which were infected with *cagA*^+^*/vacA*^+^*/*three ORFs^+^ strains (813.1 ± 96.05 pg/ml, n = 5; Fig. 5) than the cells infected with *cagA*^−^*/vacA*^−^*/*three ORFs^+^ strains (492.4 ± 54.79 pg/ml, n = 5; Fig. 5), whereas the cells infected with triple negative strains (*cagA*-*/vacA*-*/*three ORFs) recorded the lowest IL-8 induction (94.28 ± 20.45 pg/ml, n = 5; Fig. 5). Here, *vacA* s1m1 and *vacA* s2m2 alleles are taken as *vacA*^+^ and *vacA*^−^, respectively because *vacA* s2m2 allele is nonfunctional.

### Three ORFs positive strains trigger more apoptosis in AGS cells

**Fig. 6:**
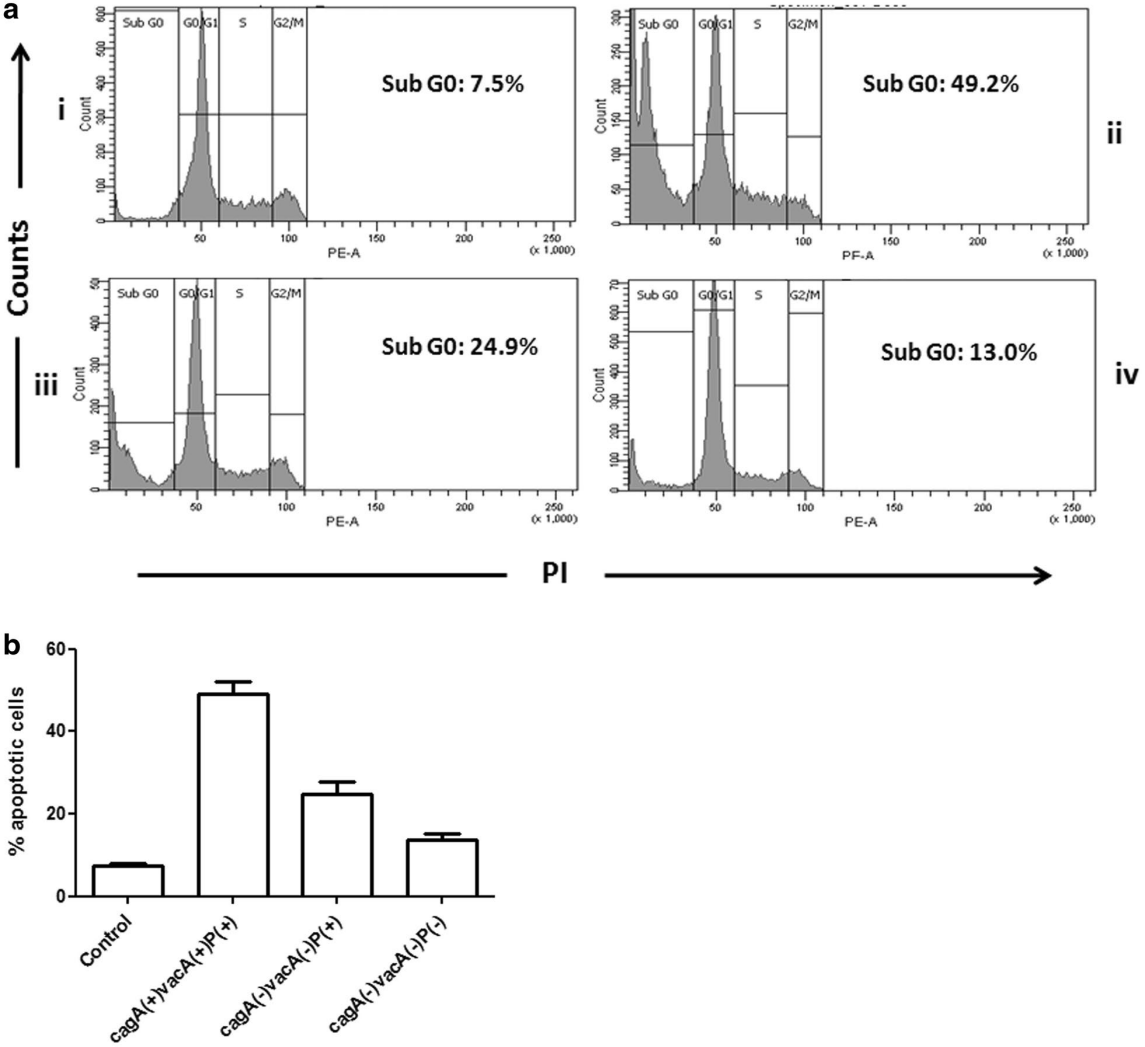
Importance of *H. pylori* plasticity region in cell cycle analysis of AGS cells.** a** Cell cycle analysis of AGS cells *i* worked as control) co-cultured with different genotypic variant i.e. *ii* *cagA*(+)*vacA*(+)P(+); *iii* *cagA*(−)*vacA*(−)P(+) and *iv* *cagA*(−)*vacA*(−)P(−). *H. pylori* strains for 24 h (MOI is 100), stained with propidium iodide, processed and analysed by flow cytometry. These figures are representative profile of at least three experiments.** b** Graphical representation of  % apoptotic cells (Sub G0 phase) infected with same group of strains were expressed as mean ± SEM. *vacA* positive and negative denote the *vacA* s1m1 and *vacA* s2m2 alleles, respectively

The cell cycle analysis with propidium iodide reveals distribution of cells in three major phases of the cell cycle (G1, S and G2/M) and makes it possible to detect unhealthy cells with fractional DNA content. The cells in the sub-G0 phase represent apoptotic cells. After co-culturing the AGS cells with *H. pylori* for 24 h significantly (*P* < 0.05) higher amount of apoptotic cell death was found when the cells were infected with *cagA*^+^*/vacA*^+^*/*three ORFs^+^ strains (49.13 ± 3.002 %, Fig. 6) than the cells infected with *cagA*^−^*/vacA*^−^*/*three ORFs^+^ strains (24.78 ± 2.936 %, Fig. 6) and infection with the triple negative strains (*cagA*^−^*/vacA*^*−*^*/*three ORFs^−^) caused the least apoptotic cell damage (13.63 ± 1.52 %, Fig. 6).

### Strains harboring three ORFs cause more induction of caspase-3 in AGS cells

**Fig. 7:**
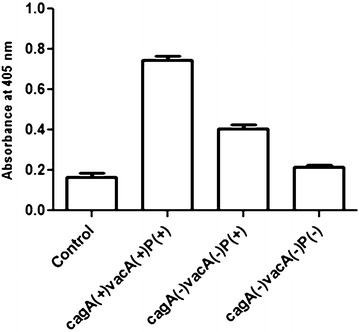
Spectrophotometric analysis of caspase 3. Caspase analysis of AGS cells (controls) co-cultured with different genotypic variant i.e. *cagA*(+)*vacA*(+)P(+);*cagA*(−)*vacA*(−)P(+) and *cagA*(−)*vacA*(−)P(−) *H. pylori* strains for 24 h (MOI is 100), cells were then lysed and supernatant was used for colorimetric assay of caspase-3 activity as described in "Methods". Data are expressed as mean ± standard error of mean (SEM) of 3 experiments in duplicates. *vacA* positive and negative denote the *vacA* s1m1 and *vacA* s2m2 alleles, respectively

We also assessed the effects of combined effects of these three ORFs regarding apoptosis via the level of caspase-3 activity. Cleavage of caspase-3 is a regular concluding pathway in caspase-mediated cell death initiated by any agents. Activation of Caspase-3 by *H. pylori* was determined by measuring cleavage of the colorimetric substrate DEVD-pNa as described in methods. We measured the degree of caspase-3 activity in *H. pylori* infection by using biochemical assays of lysates from cells infected with the aforesaid group of strains having particular genotype (n = 3 in each group). As shown in Fig. 7, caspase-3 activity was significantly higher (*P* < *0.05*) after 24 h of infection in *cagA*^+^*/vacA*^+^*/*three ORFs^+^ strains infected AGS cells compared to the cells infected with *cagA*^−^*/vacA*^−^*/*three ORFs^+^ strains, followed by triple negative strains (*cagA*^−^*/vacA*^−^*/*three ORFs^−^).

## Discussion

Identification of a disease-specific *H. pylori* virulence factors prognostic of the consequence of infection still remains elusive. Although a number of putative virulence factors of *H. pylori* has been reported (e.g., the *cag-*PAI, *vacA*, *babA*, and *oipA)* to be associated with the clinical outcomes of *H. pylori* infection, they fail to answer the entire process of disease manifestation in all geographical areas in the world, especially in the context of Asian countries. Prevalence of two most studied virulence determinants namely; *cagA and vacA* of *H. pylori* strains isolated from DU and NUD patients in Asian countries do not show any significant variations. So, studies searching on new putative virulence factors in *H. pylori* are still essential. Under this condition, there has been considerable recent curiosity in the plasticity region, whose gene content varies among different isolates and may play important roles in the pathogenesis of *H. pylori*-associated diseases. Using a comparative genome analysis, Fischer et al. recently concluded that major parts of the *H. pylori* plasticity zones reported earlier should in fact be considered as mobile genetic elements with conserved gene content, rather than regions of genome plasticity. They have also suggested that the high prevalence and wide distribution of these regions throughout all *H. pylori* populations might provide an as yet unknown fitness benefit to their hosts.

Studies indicated that Novel protein antigen (JHP0940) from plasticity region in *H. pylori* elicited strong and significant levels of tumor necrosis factor alpha and interleukin-8 in human macrophages [26]. Moreover, according to Kim et al. [27] JHP0940 is a catalytically active protein kinase that translocates into cultured human cells and with that kinase activity is capable of indirect upregulation of NF-κB p65 phosphorylation at Ser276. These observations might suggest the putative role of *jhp0940* in chronic gastric inflammation and, possibly, the various other outcomes of *H. pylori* infection, including gastric cancer.

The prevalence of *jhp0940* in Western and East Asian isolates has been reported as 17.2 and 23.5 %, respectively. Studies with Brazilian patients indicated that the *jhp0940* gene was found in only three of 200 *H. pylori* strains tested [28]. Other studies demonstrated that 62 % (71 from 114 strains) and 53.1 % of the *H. pylori* strains isolated from Pakistan and Mexico were positive for *jhp0940,* respectively [15, 29]. But our study showed that only 13.3 % of the *H. pylori* isolates from India were positive for *jhp0940*. According to Occhialini et al. [17]., in a Costa Rican population about 41.2 % isolates from gastric cancer patients were *jhp0940* positive, where all the isolates from gastritis patients were found negative (*P* < 0.0006). Study from Pakistan also demonstrated that gastric ulcer (GU) was more significantly associated with *jhp0940* (17 patients, 77 %; *P* = 0.003) than with gastritis (14 patients, 39 %) [15]. In contrary, Sugimoto et al. [22] reported that *jhp0940*-positive Western isolates were significantly associated with absence of gastric ulcer or duodenal ulcer [0.21 (0.05–0.94) and 0.31 (0.12–0.78], respectively). Our study is partially in accord with this Western finding as *jhp0940* is almost two times higher in NUD than the DU in Indian population although the difference was not significant. Our results might propose that *jhp0940* has a protective effect on gastroduodenal diseases although findings from various research groups from different parts of the world produced contradictory reports regarding the prevalence as well as its effect. The absence of *jhp0940* was supported by a probability of deletion because there are reports showing deletion of some genes from evolved *H. pylori* strains isolated from advanced stages of gastric diseases when chronic atrophic gastritis progressed to gastric cancer in the same patient over a time of four years [30]. Thus, it is possible that due to high rate of evolution of the bacteria, *jhp0940* might get deleted from *H. pylori* strains during progression of gastritis to duodenal ulcer. However, there are no reports on how the bacteria modulate these types of deletions within a single host during disease progression and to justify such rapid evolution.

Studies from Turkey, Costa Rica, and Netherlands reported that the prevalence of *jhp0945* status was parallel between *H. pylori* from patients with peptic ulcer and that from patients with gastritis but the sample size was small in those studies [17]. Another comprehensive study by Sugimoto et al. [22] among 300 *H. pylori* isolates from Western population presented a significant association between *jhp0945*-positive isolates and gastric ulcer, duodenal ulcer, and gastric cancer. Same study also reported that the *jhp0945* status was associated with an increased risk of gastric ulcer (odds ratio (OR) 2.58, 95 % CI 1.06–6.27) in East Asia using univariant analysis. Our results are consistent with this finding and showed that *jhp0945* status is significantly associated with DU, which supports the finding of Sugimoto et al. [22].

The prevalence of *jhp0947* in East Asian isolates was only 5.5 % [22]. In 2000, Occhialini et al. [17] suggested about a more frequent distribution of *jhp0947* in gastric cancer isolates (64.7 %) than in those from gastritis patients (34.6 %). Moreover, Santos et al, [28] described that the presence of the *jhp0947* remained associated only with gastric cancer (OR 2.94, 95 % CI, 1.86–4.64) and with duodenal ulcer disease (4.84, 2.13–10.96) using multivariate analysis. Moreover, Yakoob et al. [15] found a significant association of *jhp0947* with chronic active inflammation and multivariate analysis demonstrated that the ORF was associated with DU in Pakistan and our study also showed that the *jhp0947* status was associated with a significantly increased risk of duodenal ulcer.

One study in a Dutch population established that the presence of *jhp0949* was significantly associated with duodenal ulcer compared to gastritis [31]. Another study reported that 83.7 % of the *H. pylori* strains isolated from Mexican children was positive for *jhp0949* [29]. Sugimoto et al. [22] reported that there were no significant association between the development of gastroduodenal disease and the status of *jhp0949* in East Asian and Western population. But the present study in Indian population demonstrated significant association in the prevalence of *jhp0949* between patients with NUD and those with *H. pylori* related duodenal ulcer. These differences may well reflect the geographic variation of the *H. pylori* isolates used in various studies. Further, this study also displayed that IL-8 production and apoptotic cell death were significantly higher in *H. pylori* strains containing *jhp0945*, *jhp0947* and *jhp0949* than the strains lacking those genes. Together, these results may emphasize that the presence of *jhp0945*, *jhp0947* and *jhp0949* were significantly associated with symptomatic expressions whereas *jhp0940* seems to be negatively associated with the disease status.

## Conclusions

In conclusion, this was the first study in India to assess the relationship between plasticity region genes and clinical manifestations. The findings of the study suggest that *jhp0945*, *jhp0947* and *jhp0949* could be useful prognostic markers for the development of peptic ulcer in Indian population whereas *jhp0940* seems to be negatively associated with the disease.

## Methods

### Collection of Biopsy samples

A total of 171 adult patients of both sexes (aged between 23 and 71 years) with gastric complaints were subjected to endoscopy at the hospital of the Institute of Post Graduate Medical Education and Research, Kolkata, and St. John’s Medical College Hospital, Bangalore, India during the year 2007–2009. Complete patient’s history was noted, and a physical examination of each subject was performed before endoscopy. The NICED Ethical committee had approved the study. The record regarding the patient information was kept blind during the experimental procedures and the disease status was decoded during the data analysis. Two biopsies, one from antrum and the other from corpus of the stomach, were taken during endoscopy, from each individual. Biopsies obtained in 0.6 ml of Brucella broth (Difco Laboratories, Detroit, MI) with 15 % glycerol were transported to the laboratory in ice-cold condition and were stored at −70 °C until culture.

### *H. Pylori* culture

In the laboratory, Brucella broth containing the specimen was vortexed for 2 min and 200 µl of the mixture was streaked on Petri plates containing brain heart infusion (BHI) agar (Difco Laboratories) supplemented with 7 % sheep blood, 0.4 % IsoVitaleX, amphotericin B (8 µg/ml) (Sigma Chemicals Co., St. Louis, MO), trimethoprim (5 µg/ml), vancomycin (6 µg/ml) (Sigma Chemicals) and Nalidixic acid (8 µg/ml) (all from Sigma). Plates were incubated for 3–6 days at 37 °C in a double gas incubator (Heraeus Instrument, Germany) which maintains an atmosphere of 85 % N_2_, 10 % CO_2_, and 5 % O_2_ [32]. The *H. pylori* colonies were identified by their typical colony morphology, appearance on Gram staining and positive reactions in urease, catalase and oxidase tests along with the urease PCR. Bacteria were sub-cultured at 37 °C on the above medium and under the same microaerophilic condition.

### Extraction of genomic DNA

Cells were harvested from the culture plates and washed with phosphate-buffer saline (pH 8.0) followed by centrifugation at 3000 rpm for 1 min. The pelleted cells were resuspended in 540 µl of TE buffer (10 mM Tris–HCL, 1 mM EDTA), 60 µl of 10 % Sodium dodecyl sulfate (SDS) (Sigma) and 9 µl of Proteinase K (20 mg/ml) (Invitrogen, Carlsbad, CA), mixture was incubated at 50 °C for 1 h followed by addition of 100 µl of 5 M NaCl, 80 µl of 10 % CTAB solution and then again incubated at 65 °C for 10 min. The DNA was extracted according to the standard phenol–chloroform-method [33].

### PCR amplification

PCR amplification was performed in a final volume of 20 µl containing template DNA (2–20 ng), 2 µl of 10× Buffer (Roche, Germany), 2.5 mM dNTPs (Roche) and 10 pmol of corresponding primers (Table 2) in the presence of 1U of Taq DNA Polymerase (Roche). The cycling program has the following condition: initial denaturation at 95 °C for 3 min followed by 30 cycles of denaturation at 94 °C for 1 min, annealing at 55 °C for 1 min, extension at 72 °C for 1 min, and final extension at 72 °C for 7 min. Genomic DNA from the strain J99 and 26695 were included as positive and negative control respectively. The PCR products were analyzed by 1.5 % agarose gels (containing 0.5 µg of ethidium bromide per ml) in 1X TAE buffer. Gels were scanned under UV light and analyzed with Quantity One software (Bio-Rad, Hercules, CA). The size of product was confirmed by using molecular weight marker.

**Table 2:** Primers used in this study

Primer	Sequence (5′-3′)	Amplicon (bp)	Reference
jhp0940F	GAAATGTCCTATACCAATGG	600	[22] [22]
jhp0940R	CCTAAGTAGTGCATCAAGG		
jhp0945F	ACTCCAGCCAGTATTGTAAA	246	[22] [22]
jhp0945R	TTCTTGCGAGTTAGGATTGG		
jhp0947F	GATAATCCTACGCAGAAC	650	[22] [22]
jhp0947R	GCTAAAGTCATTTGGCTGTC		
jhp0949F	ATAGGAGTGGGTGCTTACTT	788	[22] [22]
jhp0949R	AGCAACAACAAAGGCATGTA		
ureBF	CGTCCGGCAATAGCTGCCATAGT	480	[34] [34]
ureBR	GTAGGTCCTGCTACTGAAGCCTTA		
CagA5CF	GTTGATAACGCTGTCGCTTCA	350	[25] [25]
CagA3CR	GGGTTGTATGATATTTTCCATAA		
Lunil1	ACATTTTGGCTAAATAAACGCTG	550	[25] [25]
R5280	CCAACGTGCGTAAAAGGGAATTAG		
vacAsF	ATGGAAATACAACAAACACAC	s1-259/s2-286	[35] [35]
vacAsR	CTGCTTGAATGCGCCAAAC		
vacAmF	CAATCTGTCCAATCAAGCGAG	m1-567/m2-642	[35] [35]
vacAmR	GCGTCAAAATAATTCCAAGG		

### IL-8 assay

All the bacterial strains were cultured in 7 % serum containing BHIA plates for 24 h at 37 °C under microaerophilic conditions. In order to obtain in vitro IL-8 secretion from gastric epithelial cells, AGS **(**human gastric adenocarcinoma cell line) cells were plated (2.5 × 10^5^cells/ml) into 24 well plates and cultured for 24 h. *H. pylori* (multiplicity of infection (MOI) of 100) were added to cultured cells. After 8 h. of infection, IL-8 levels in the supernatant were assayed in duplicate three times using a commercially available specific ELISA kit (Genetix, India) following the manufacturer’s protocols.

### Cell cycle analysis

AGS cells (1 × 10^6^cells/ml in each well) were infected with exponentially growing *H. pylori* culture. After 24 h of infection cells were fixed in 70 % chilled ethanol and were kept at 4°C for further analysis. Prior to analysis cells were washed in 2 % fetal bovine serum (FBS) containing PBS (pH 7.4) and the cell pellets were stained with propidium iodide (50 µg/ml) containing DNase-free RNase (0.1 mg/ml). Cells were then acquired on flow cytometer and the data was analyzed in FACS Diva (Becton–Dickinson, USA) software.

### In vitro caspase-3 activity assay

The AGS cells were plated (2.5 × 10^6^ cells/ml in each plate) into petriplate (60 mm dias) to perform this experiment and cultured for 24 h. The cells were then infected with one day old *H. pylori* culture (multiplicity of infection [MOI] of 100). After 24 h of infection the AGS cells were collected by centrifugation at 1000×*g* for 10 min at room temperature, which were then washed twice with PBS. A suspension of these cells were prepared then in lysis buffer at a density of 10^7^ cells/ml and kept on ice for 10 min. The cell debris was discarded by centrifugation at 16,000×*g* for 5 min at 4 °C, and the cell supernatant was then used for the colorimetric assay of caspase-3 activity using commercially available kit (Abcam, Cambridge, UK). Protein concentrations were measured using the Bio-Rad protein assay according to the manufacturer’s protocols.

### Statistical analysis

Each experiment was performed at least thrice in duplicates and results expressed as mean ± standard error of the mean (SEM). Statistical analysis was done by T test and ANOVA (wherever applicable). Univariate analysis was done to determine Odds ratio (OR) and Confidence interval (CI). Calculations were done using Graph Pad Prism software (version 5, Graph Pad Software Inc, USA) and *P* values <0.05 were considered to be significant.
